# Thunderstorm charge structures producing gigantic jets

**DOI:** 10.1038/s41598-018-36309-z

**Published:** 2018-12-27

**Authors:** Levi D. Boggs, Ningyu Liu, Jeremy A. Riousset, Feng Shi, Steven Lazarus, Michael Splitt, Hamid K. Rassoul

**Affiliations:** 1 Aerospace, Physics and Space Sciences Department, Florida Institute of Technology, Melbourne, FL USA; 2 Department of Physics and Space Science Center, University of New Hampshire, Durham, NH USA; 3 Ocean Engineering and Marine Sciences, Florida Institute of Technology, Melbourne, FL USA; 4 College of Aeronautics, Florida Institute of Technology, Melbourne, FL USA

## Abstract

Gigantic jets are atmospheric electrical discharges that propagate from the top of thunderclouds to the lower ionosphere. They begin as lightning leaders inside the thundercloud, and the thundercloud charge structure primarily determines if the leader is able to escape upward and form a gigantic jet. No observationally verified studies have been reported on the thundercloud charge structures of the parent storms of gigantic jets. Here we present meteorological observations and lightning simulation results to identify a probable thundercloud charge structure of those storms. The charge structure features a narrow upper charge region that forms near the end of an intense convective pulse. The convective pulse produces strong storm top divergence and turbulence, as indicated by large values of storm top radial velocity differentials and spectrum width. The simulations show the charge structure produces leader trees closely matching observations. This charge structure may occur at brief intervals during a thunderstorm’s evolution due to the brief nature of convective pulses, which may explain the rarity of gigantic jets compared to other forms of atmospheric electrical discharges.

## Introduction

Nearly all gigantic jets have been found to originate in tall (14–18 km altitude), intense thunderstorms featuring overshooting tops that form in maritime tropical environments^1–9^. There has been one documented case of a gigantic jet emerging from a low topped (6.5 km altitude) winter thunderstorm over the Mediterranean Sea, but it also had an overshooting top as the environmental tropopause was near 6 km altitude^10^. When the emerging location of gigantic jets at the storm top can be accurately determined, they appear to escape from or near the convective core of the thunderstorm^2,3,5–8^. Often gigantic jets occur during or near the end of a convective ‘pulse’, which corresponds to a period of rapid thunderstorm intensification^4,6,8^. It has also been found that gigantic jets escape the thundercloud along an axis that marks the center of their parent storm’s divergent outflow^8^ and that some of these storms form in environments with large horizontal wind speeds near the altitudes of their respective thundercloud tops^8,10,11^.

Because gigantic jet observations are rare^12,13^, it is nearly impossible to obtain *in-situ* measurements (e.g., balloons, etc.) to understand the charge structure producing those unusual electrical phenomena. Our current knowledge of the charge structure is mainly obtained from modeling studies and analysis of meteorological and lightning data. Previous modeling studies have shown that for a normal polarity thunderstorm that has a classic tripolar charge structure (vertically stacked regions of upper positive, middle negative, and lower positive charge), gigantic jets are initiated between the middle negative and upper positive charge regions as a normal intracloud flash, with the negative leader subsequently escaping the upper positive charge^14,15^. This result is consistent with the observed gigantic jets being predominately of negative polarity and transferring negative charge to the ionosphere^1–5,7,9,16^. In order for the negative leader to escape the cloud, the upper positive charge must be weakened - likely by mixing with the upper negative screening charge layer^14,15^. This creates a charge imbalance between the main thundercloud charge regions, enabling the leader to escape.

The charge structures used in those modeling studies were not formulated from direct observations of gigantic jet producing convection, but were assumed to resemble a classic tripolar charge structure^17,18^. They featured a wide, weakened upper positive charge region over a similarly sized middle negative charge region^14,15^, augmented by a small lower positive charge region. The simulations were conducted with the top boundary set right above the cloud top, and their purpose was to determine if a lightning leader from a normal intracloud flash could escape the upper charge region. They did not answer where this leader would propagate once it left the upper charge and whether it would form a bolt-from-the-blue, jet, or gigantic jet. These studies, however, successfully demonstrated that a charge imbalance inside the thundercloud is required for a leader to escape.

Past observational studies have analyzed meteorological data^6,8^ of gigantic jet producing storms and analyzed the parent lightning^16^ associated with gigantic jets. Gigantic jet producing convection was found to have moderate to high convective available potential energy values (1200–3500 J kg^−1^) and 10 dBZ radar reflectivity values reaching 14–17 km altitude^6,8^. The storms analyzed by Meyer *et al*.^6^ had peak altitudes of very high frequency lightning data and radar reflectivity near the times of the gigantic jets, which is consistent with the storms undergoing a convective pulse. Lazarus *et al*.^8^ found that storm top turbulence, as inferred from eddy dissipation rate^19^, was at a maximum when the gigantic jets occurred. The gigantic jets also escaped along the center axis of divergent outflow near storm top. Lu *et al*.^16^ found the parent lightning as observed by very high frequency networks resembled an ordinary intracloud flash between the middle negative and upper positive charge regions, but with an ‘attempted’ bolt-from-the-blue discharge^16^ preceding the gigantic jets.

This paper attempts to identify the thundercloud charge structure that produces gigantic jets, and aims to determine how a lightning discharge develops after it leaves the thundercloud charge. The charge structure is formulated by combining lightning and radar data analysis of gigantic jet producing convection with an emphasis on thunderstorm charge structure, and from lightning simulations performed using a three dimensional probabilistic model. The probabilistic lightning simulations are performed over a larger simulation domain than those used in previous studies to study where the escaped lightning leader propagates. Simulations for two other thunderstorm charge structures, which are either proposed by previous modeling studies or possibly formed during the parent storm of gigantic jets, are also performed to identify the most likely charge structure that produces gigantic jets. Finally, this study seeks to understand the meteorological processes that form this charge structure.

## Results

### Radar and lightning observations

**Figure 1:**
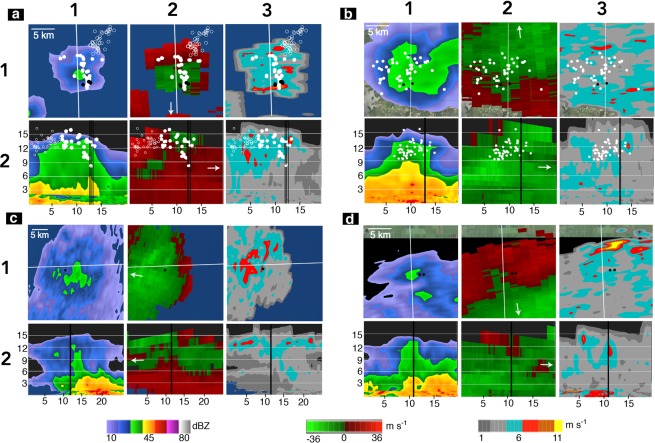
Common features of four gigantic jet producing storms. The top rows of each panel show horizontal elevation angle scans of base reflectivity, radial velocity, and spectrum width of the upper regions of the thundercloud (12–15 km). The bottom rows show vertical cross sections, along the white lines in the top row, for each radar variable. Radar and lightning data for the (**a**) Northeast FL storm on 28 September 2010^16^ (**b**) South OK storm on 09 September 2010^16^ (**c**) Southeast FL storm on 03 August 2013^7,8^ and (**d**) Southcentral FL storm on 12 September 2014^11^. VHF lightning mapping data of the discharge activity in the upper positive charge region leading to each gigantic jet is shown (when available) as white circles and NLDN IC events as black circles or black vertical lines. The attempted bolt-from-the-blue is shown as open white circles^16^. The white arrows in column 2 of each panel denote the direction pointing to the radar. Distance scales are listed in km.

To better understand the common thunderstorm features near cloud top during gigantic jets, base reflectivity, radial velocity, and spectrum width derived from Weather Surveillance Radar 88-Doppler (WSR-88D) radar scans were analyzed for four gigantic jet producing thunderstorms (Fig. 1 and Table 1). Reflectivity is a measure of the power scattered back to the radar from the target, radial velocity is the inbound/outbound velocity along a radial path extending from the radar, and spectrum width is a measure of the radial velocity spectrum in a radar bin. Spectrum width can be used as a proxy for turbulence, where large spectrum width is associated with increased turbulence^20,21^. The parent thunderstorm dates, approximate times, and locations are listed in Table 1. Some of the storms analyzed here were studied in detail before concerning their meteorological features and lightning activity, as obtained by radar measurements and lightning detection data^6,8,16^. Here we focus on analyzing the meteorological and lightning data that gives information of the temporal and spatial properties of the charge structure of those storms.

**Table 1:** Overview of the gigantic jet storms shown in Fig. 1.

Date	Time (UTC)	Event (location)	RV Δ(m/s)	Max SW (m/s)
September 2010	07:28	South OK, USA	38	11
September 2010	11:01	Northeast FL, USA	35	10
August 2013	04:11	Southeast FL, USA	55	9
September 2014	06:59	Southcentral FL, USA	26	11

The radial velocity differential (RVΔ) is defined as the absolute value of the maximum outbound minus the maximum inbound radial velocities. The radial velocity differentials and maximum spectrum width (Max SW) values are taken from a radial at storm top passing through the region of largest reflectivity.

All storms had reflectivity values greater than 30 dBZ at high altitudes (>12 km) and tall thundercloud tops (Fig. 1a–d, column 1). Strong horizontally diverging winds near the thundercloud top were present in all cases (Fig. 1a–d, column 2), with radial velocity differentials of 26–55 m s^−1^ (Table 1). The vertical columns of high reflectivity were collocated with the centers of divergent outflow. Large values of spectrum width (9–11 m s^−1^) were horizontally displaced from and just outside the convective core (Fig. 1a–d, column 3). These large values of spectrum width indicate turbulent mixing^20^ near the thundercloud top, and are indicators of where upper negative screening charge may be mixed with upper positive charge^11^. These values are particularly large, as past studies have shown that spectrum width values greater than 4 m s^−1^ are considered turbulent^22^. Lastly, the National Lightning Detection Network intracloud (NLDN IC) events and very high frequency (VHF) lightning sources associated with each gigantic jet were located near the axis of divergent outflow and near the convective core - except for the attempted bolt-from-the-blue (discussed in Lu *et al*.^16^ - open white circles in Fig. 1a).

**Figure 2:**
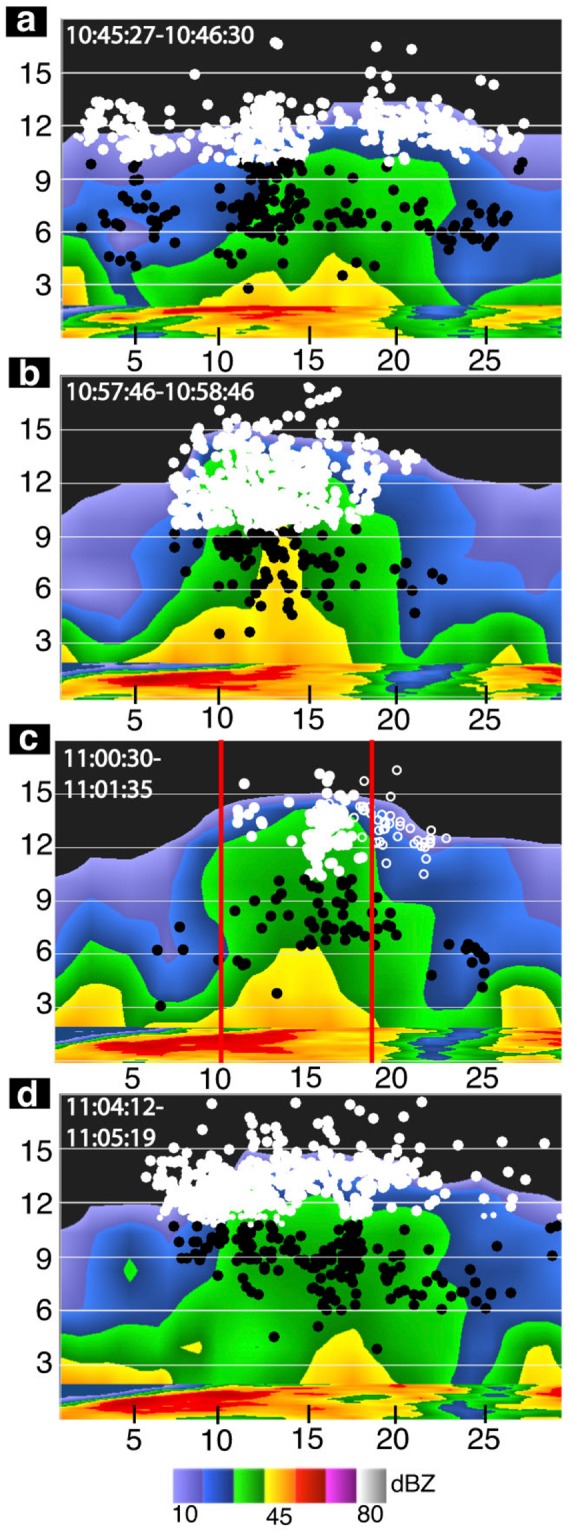
Charge structure evolution for the Florida gigantic jet on 28 September 2010. VHF inferred charge structure for the (**a**) pre-pulse (**b**) initial pulse (**c**) final pulse (gigantic jet) and (**d**) post pulse times. The white circles denote upper positive charge and the black circles denote middle negative charge as inferred from the VHF data. The VHF data is shown for the times to the upper left (also in Table 2). The attempted bolt-from-the-blue is shown as open white circles^16^. The red vertical lines in (**c**) denote the edges of large spectrum width values shown in Fig. 1a, column 3. Distance scales are listed in km.

The charge structure producing gigantic jets is found from a combination of different radar variables, lightning data, and lightning simulations, but information about the charge structure is first obtained from re-analyzing available VHF lightning mapping data at different periods of the storms. The evolution of the VHF inferred charge structure for the 28 September 2010 Florida gigantic jet is investigated at four time periods, each about one minute long (Fig. 2 and Table 2). This case is chosen as it is closest to the VHF mapping system (about 60 km away). The details of determining the thundercloud charge structure are described in the Methods section. This storm underwent a convective surge or pulse near the time of the gigantic jet, as identified by Meyer *et al*.^6^ and supported by Fig. 2 and Table 2. The VHF charge analysis times are: before the convective pulse (pre-pulse), near the beginning of the convective pulse (initial pulse), near the end of the convective pulse and during the time of the gigantic jet (final pulse), and after the convective pulse (post-pulse). The specific time periods analyzed are shown in Table 2, and each period corresponds to a panel in Fig. 2. A similar analysis was completed for the 09 September 2010 Oklahoma thunderstorm that produced two gigantic jets^6,16^ and the 03 August 2013 Florida thunderstorm that produced four gigantic jets^7,8^, both of which had a charge structure evolution similar to the 28 September 2010 Florida thunderstorm (see Supplementary Information).

**Table 2:** Statistics describing the upper positive charge region for the Florida thunderstorm on 28 September 2010.

	Time (UTC)	ΔX (km)	$\overline{Z}$ (km)
Pre-Pulse	10:45:27–10:46:30	25.5	11.5
Initial Pulse	10:57:46–10:58:46	5.8	11.7
Final Pulse (GJ)	11:00:30–11:01:35	4.9	13.2
Post-Pulse	11:04:12–11:05:19	12.7	12.9

ΔX represents one standard deviation about the mean of the VHF sources in the azimuthal direction, and $\overline{Z}$ represents the mean altitude of VHF sources.

The thunderstorm that produced the 28 September 2010 Florida gigantic jet had a wide, diffuse upper positive charge region over a similarly sized middle negative charge region before the convective pulse (Fig. 2a). The large horizontal extent of the upper positive charge is indicated by the large azimuthal variation of VHF sources (ΔX), which was 25.5 km (Table 2). The mean altitude of VHF sources ($\overline{Z}$) in the upper positive charge region was 11.5 km altitude. The charge configuration during this time closely resembled a classic tripolar thunderstorm charge structure, but with a very small or nonexistent lower positive charge region as indicated by the lack of VHF sources at lower altitudes. Consistently, there were only three NLDN reported negative cloud-to-ground discharges in the time spanning fifteen minutes before the gigantic jet. There was a relatively weak divergence couplet present at storm top during this time, with a radial velocity differential of 29 m s^−1^ (not shown in Table 2). During the onset of the convective pulse, the number of VHF sources in the upper positive charge increased, and the upper positive charge climbed higher in altitude (Fig. 2b). The majority of VHF sources were contained within the relatively narrow reflectivity column (>30 dBZ). The intensity of the pulse was reflected by the altitude of the 45 dBZ echo, which reached a local maximum during this time (about 10 km, compared with 5 km before and after the pulse). The radial velocity differential at storm top increased to 38 m s^−1^. Near the end of the convective pulse and during the gigantic jet (Fig. 2c), the upper positive charge reached its highest altitude ($\overline{Z}$ of 13.2 km) and became very narrow, with a ΔX of 4.9 km. The majority of the VHF sources were confined within the boundaries of maximum spectrum width (marked as vertical lines in Fig. 2c) surrounding the reflectivity column (>30 dBZ), and there was a significant decrease in the number of VHF sources. The radial velocity differential at storm top reached its maximum during this time, with a value of 40 m s^−1^. The open white circles shown in Fig. 2c were from the ‘attempted bolt-from-the-blue’^16^, which was part of the parent gigantic jet flash. After the convective pulse, the upper positive charge widened again (ΔX of 12.7 km) and began to subside ($\overline{Z}$ of 12.9 km). However, the radial velocity differential at storm top remained large (39 m s^−1^) until the next radar volume scan (four minutes later), when the radial divergence couplet disappeared altogether.

### Probabilistic lightning simulations

**Figure 3:**
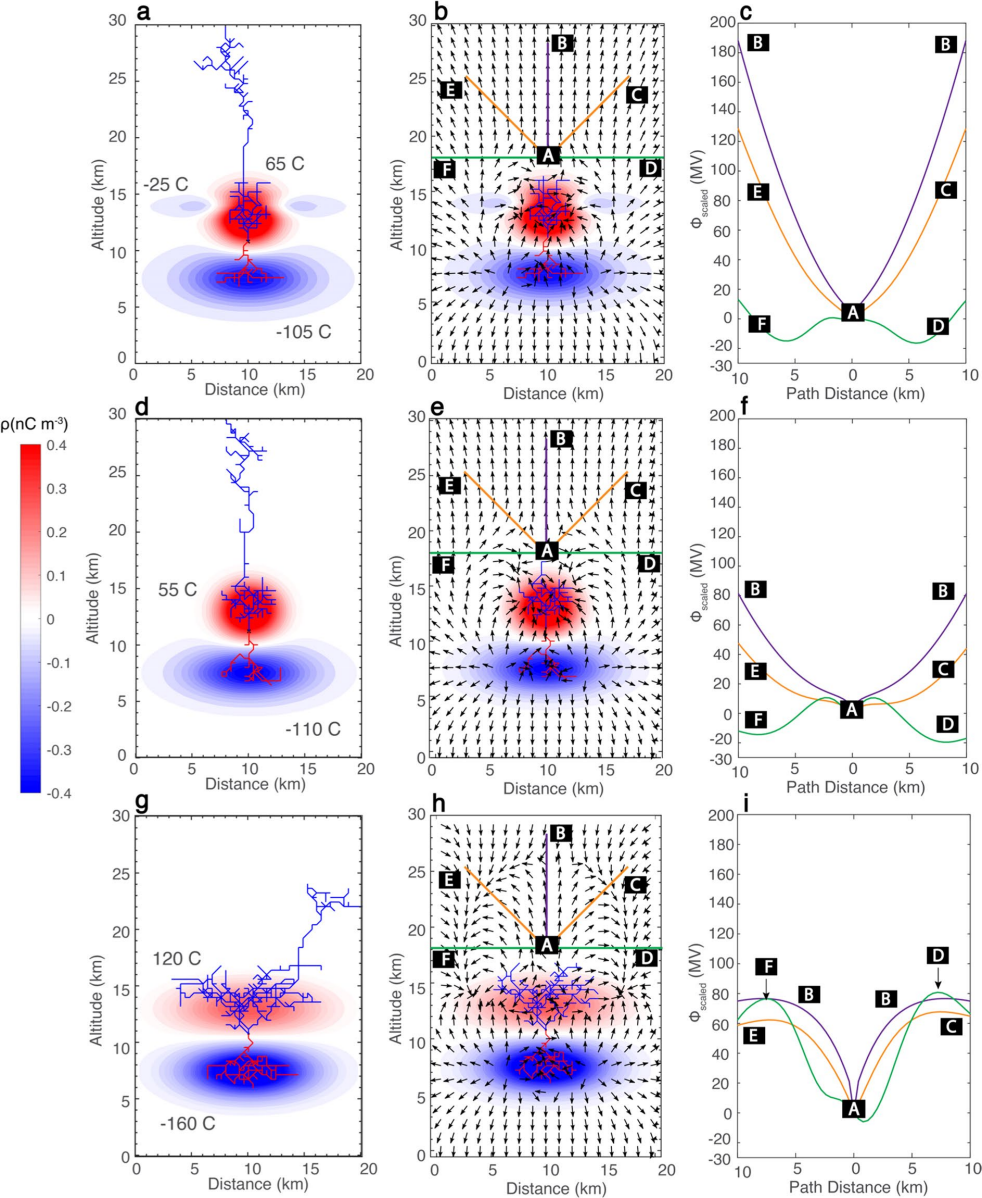
Simulations of potential gigantic jets. (Left column) Simulated discharge trees overlaid on Gaussian thunderstorm charge structures. Positive (negative) charges and leaders are colored in red (blue). Charge amounts are in Coulombs. (Middle column) Direction of −***E*** overlaid on the thunderstorm charge structures as the negative leaders escape the upper charge regions. Also overlaid are paths used for calculation of *ϕ*_*scaled*_. (Right column) *ϕ*_*scaled*_ for each path denoted in the panels of the middle column.

Simulations using a three dimensional probabilistic lightning model^14,15^ were performed in conjunction with the data analysis discussed above in order to find the charge structure of gigantic jet producing storms (Fig. 3). Three charge structures were tested and simulations for each charge structure were run a total of ten times to find the discharge patterns that were most prevalent for each charge structure, so we would not report on outliers. If the discharge reaches the top boundary of the simulation domain, it is categorized as a possible gigantic jet. It should be noted that the model does not simulate the temporal evolution of the discharges, but the spatial characteristics of the discharges are simulated, similar to previous studies^14,15,23,24^.

Two charge structures feature narrow upper positive charge (Fig. 3a,d), but one has a distribution of upper negative screening charge around the top of the upper positive charge (Fig. 3a). The charge structure with upper negative screening charge is considered because it is probable that upper negative screening charge exists around the highest cloud tops of convective cells that produce negative gigantic jets. Considering the relaxation time at storm top is very short (about 15 s at 15 km altitude^15,25^), negative screening charge can form quickly at high storm tops. But, the strong storm top diverging winds should push away the screening charge from the center axis of the convective core, which would make a ‘hole’ in the upper negative screening charge. In nature this screening charge is likely not perfectly symmetric, but for the sake of simplicity, perfect axial symmetry is assumed. The altitudes and dimensions of the upper positive and middle negative charge regions in Fig. 3a,d are set according to the lightning and radar data in the previous section during the time of the gigantic jets. A charge structure with a wide upper positive charge region, similar to previous modeling studies of escaped leaders^14,15^ is also considered (Fig. 3g), which models the charge structure shown by Fig. 2a,d. Lower positive charge is likely very small immediately preceding and during gigantic jets^6,11^, as shown in Fig. 2 and discussed above, and therefore are not included in the charge structures. However, simulations with lower positive charge were also completed (see Supplementary Information), and the results indicate that the inclusion of small lower positive charge does not change the conclusions of this study. Considering the convective systems producing gigantic jets are very wide (about 40–60 km for the storms presented here), the lateral cloud edges are far from the parent GJ flash, so the lateral screening charges are not included in the simulations.

After specifying the geometry of the charge regions, the only key parameter left to specify the thunderstorm charge structure for simulation is the amount of charge in each region. Three constraints are used: (1) the leader discharge is initiated at the location where the electric field exceeds the threshold value for leader initiation (about 200 kV m^−1^ at ground pressure)^26–29^ by 1–10% (2) the charge densities must be less than the maximum values found from observations^30–33^ - on the order of a few tenths to a few nCm^−3^, and (3) the amount of net negative charge in the system that allows a negative leader to escape is minimized. The minimum amount of net negative charge is chosen because any additional amount of negative charge would always produce an escaped leader. This results in net charges of −65 C for Fig. 3a, −55 C for Fig. 3d, and −40 C for Fig. 3g. The minimum amount of net negative charge is different for each charge structure due to the different charge region geometries.

The discharge patterns shown in Fig. 3a,d closely resemble the parent lightning both inside and outside the thunderstorms producing gigantic jets. Within the confines of the cloud, the parent lightning has little lateral extension inside the upper positive charge region, which is consistent with the observations of the initial in-cloud discharge activity of gigantic jets^16^, as also shown by Fig. 2c. Upon exiting the thundercloud, the discharges escape as negative leaders extending upward above the convective core, similar to observations^2,3,5–8^. Finally, the discharges reach the upper boundary of the simulation domains. All ten (100%) simulations for each charge structure produced results closely resembling the discharge patterns presented in Fig. 3a,d, demonstrating the effectiveness of these charge structures to produce upward negative leaders with the capability to form gigantic jets.

The discharges simulated with a wide upper positive charge region (Fig. 3g) have large lateral extension and significant branching inside the upper positive charge region. The negative leader network in the upper positive charge extends significantly farther horizontally than the positive leader network in the middle negative charge, which contrasts with the initiating lightning observed by VHF sensors for gigantic jets^16^. When the discharge exits the upper positive charge, it bends significantly and terminates on the lateral boundary of the simulation domain. This is the dominant discharge pattern for this charge structure, with seven out of ten (70%) simulations giving such a discharge pattern. This indicates that this charge structure is conducive to propagating discharges laterally above the cloud instead of directly upward, possibly turning into a bolt-from-the-blue. The other three simulations (30%) did reach the top boundary of the simulation domain, but the discharge still showed significant bending, terminating near the upper corners of the domain. The charge structure shown by Fig. 3g also often produced multiple leaders exiting the upper positive charge, contrary to observations of gigantic jets.

Figure 3b,e,h shows the direction of −**E** overlaid on the thundercloud charge structure and discharge trees as the negative leaders exit the upper charge regions (note: in order to clearly show the direction of −**E** at every point the length of the arrow does not scale with its magnitude). We choose to show −**E** because its direction shows where a negative leader is most likely to propagate. The colored lines in these panels correspond to Fig. 3c,f,i and denote paths to calculate scaled electric potential (discussed below). In Fig. 3b,e, −**E** above the upper charge points toward the vertical symmetry axis (Distance = 10 km), and then upward, constraining the escaped negative leaders to propagate directly upward above the thundercloud. For the charge structure with wide upper positive charge (Fig. 3h), −E points outward from the vertical symmetry axis, encouraging the escaped negative leader to propagate laterally above the cloud.

Profiles of scaled electric potential (*ϕ*_*scaled*_) above the upper charge regions are shown in Fig. 3c,f,i, with the paths indicated by the colored lines in Fig. 3b,e,h. Since the lightning propagation threshold field is linearly dependent on air density^26–29^, the potential along each path is normalized to ground pressure to allow for meaningful comparisons of leader propagation along different paths. This scaled potential is calculated by

1 $${\varphi }_{scaled}={\int }_{path}\,-\,\frac{{N}_{0}}{N}{\bf{E}}\cdot {\bf{dl}}$$

with all paths beginning at the box marked A and ending at the corresponding boxed letter, with *N* (*N*_0_) the density at a given altitude (ground). Each path is 10 km in length. The path from *A* → *B* is plotted twice in panels 3c,f,i for ease of viewing. For the charge structure with narrow upper positive charge and upper negative screening charge (Fig. 3a), two minima in *ϕ*_*scaled*_ exist along the lateral paths of *A* → *F* and *A* → *D* (Fig. 3c). Considering the escaping leader is of negative polarity, it propagates towards increasing potential. Thus, the escaping leader avoids the lateral paths (*A* → *F* and *A* → *D*). The diagonal paths (*A* → *E*, *A* → *C*) and the vertical path (*A* → *B*) have large values of *ϕ*_*scaled*_, so the negative leader travels in the upward direction. But, the largest potential difference occurs along the vertical path (*A* → *B*), which prompts the escaped negative leader to propagate along the vertical symmetry axis. Figure 3f shows the scaled potential profile for the charge structure with narrow upper positive charge. The variation of *ϕ*_*scaled*_ along each path is similar to the previous case, but the vertical and diagonal paths have a smaller potential difference when compared to the charge structure of Fig. 3a. The escaped negative leader still propagates along the vertical symmetry axis where the largest potential difference is.

The charge structure with wide upper positive charge has a scaled potential profile above the cloud (Fig. 3i) that is significantly different from the charge structures with narrow upper positive charge. The largest directional derivative and largest potential difference occur for the lateral paths (*A* → *F*, *A* → *D*). Thus, the escaping negative leader propagates in the lateral direction above the cloud, instead of upward. Also, notice that the vertical and horizontal paths have a narrower spread of values in *ϕ*_*scaled*_ for this charge structure, compared with the other two cases, which increases the probability for the escaped leader to propagate in a random direction. However, the largest potential differences are along the lateral directions producing a dominant discharge pattern that extends laterally above the cloud.

**Figure 4:**
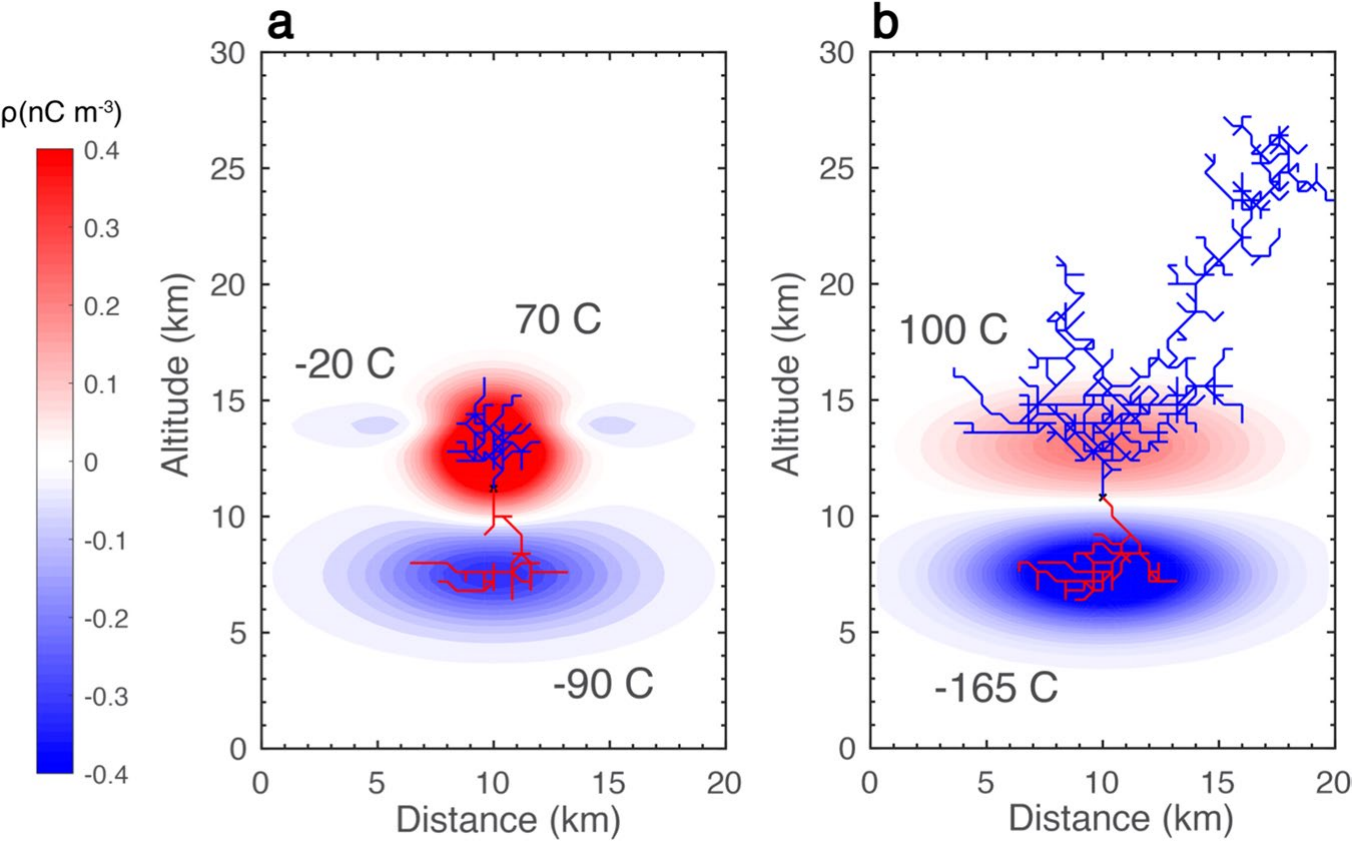
Simulations with different net charge amounts. (**a**) Same as Fig. 3a except with a net charge of −40 C. (**b**) Same as Fig. 3g only with a net charge of −65 C.

Considering the amount of net charge is different among the three cases, additional simulations were completed for the charge structures of Fig. 3a,g, only with the net charge amounts switched (Fig. 4) to investigate the effect of net charge on the discharge leader discharge tree. The charge structure in Fig. 4a does not produce any escaped negative leaders out of the additional ten simulations, forming normal IC discharges for every simulation. For the charge structure in Fig. 4b, the escaped negative leader terminates on the lateral boundary of the simulation domain. This is the dominant discharge pattern for this charge structure, which occurs for seven (70%) of the simulations and is similar to the charge structure presented in Fig. 3g. From the results shown in Figs 3 and 4, it is clear that wide, weakened upper positive charge can produce escaped leaders at a smaller amount of net charge than narrow, weakened upper positive charge, but those escaped leaders propagate in the lateral direction once they escape. Narrow, weakened upper positive charge requires more net negative charge for a leader to escape, but once a negative leader escapes, it will propagate upward. This is also true when there is more net negative charge than shown in Fig. 3a,d. Thus, whether a successfully escaped leader will propagate upward, not laterally, to potentially form a gigantic jet is primarily determined by the geometry of the charge structure and not by the amount of net charge.

## Discussion

The charge structures in Fig. 3a,d both form upward negative leaders that reach the top of the simulation domain. An approach for investigating if the escaped leader will develop into a gigantic jet is to determine if the leader tip is able to reach the jump altitude, *h*_*jump*_. The jump altitude is the altitude from which the streamers preceding the leader tip can extend all the way to the lower ionosphere. The jump altitude depends on the leader tip potential, and a larger leader tip potential gives a lower jump altitude. The ionospheric potential is about 250–300 kV^34,35^, and is much smaller than the potential of the escaped leaders. The absolute value of the leader tip potential is 24 MV (Fig. 3a) and 18 MV (Fig. 3d) when the respective leaders reach the top boundary of the simulation domain. Figure 3d of *da Silva and Pasko*^36^ indicates these values correspond to jump altitudes of 42 km and 45 km, respectively, if the streamer zone of a negative leader consists of negative streamers only. This means if the simulated leaders cease propagating upward, a negative jet is formed. It is possible the leaders continue propagating upward, and if their potentials are not reduced significantly when the leaders reach 42 km or 45 km, a gigantic jet will be formed. In addition, as discussed by *Liu et al*.^7,12^, the streamer zone of a negative leader may consist of both positive and negative streamers, so the jump altitudes for the two leaders in Fig. 3a,d can be potentially lower.

Due to the short time scale of screening charge formation at high altitudes^15,25^, the upper negative screening charge can approach the magnitude of the upper positive charge^15^, indicating the negative screening charge in Fig. 3a could potentially be much stronger. Simulations indicate (not shown) that when the upper negative screening charge in Fig. 3a is doubled to −50 C, the absolute value of the leader tip is about 60 MV when the ascending negative leader reaches the top boundary of the simulation (30 km altitude), which corresponds to a jump altitude of about 36 km (for negative streamers). This is similar to the jump altitudes of the last two gigantic jets produced by tropical depression Dorian^7^, which were about 35 km. Other things being equal under the constraints on the amount of charge in each region discussed in the previous section, the charge structure in Fig. 3a can allow accumulation of more net negative charge in the system, making it potentially easier to produce gigantic jets for this charge structure.

The wide, weakened upper positive charge structure (Fig. 3g) has a leader network above the cloud that extends significantly in the lateral direction. This charge structure assumes relatively uniform mixing of the upper negative screening charge throughout the entire volume of the upper positive charge, with a fully symmetrical configuration of the charge regions. Such a perfect symmetry likely does not occur in nature. A similar charge structure to that in Fig. 3g but with laterally displaced, weakened upper positive charge has been shown to produce bolt-from-the-blue discharges^11^, and the leader network shown in Fig. 3g resembles a bolt-from-the-blue discharge, consistent with that study.

The charge structure producing negative gigantic jets seems to be a result of the convective pulse. During the convective pulse, the radial velocity differentials and values of spectrum width at storm top reached a maximum (see Supplementary Figure S3). The maximum spectrum width values were located on the outer edge of the convective core (convective core as defined by reflectivity >30 dBZ). We theorize that the diverging winds push the negative screening charge away from the center axis of the highest cloud tops, and large turbulent eddies form around the convective core mixing negative screening charge with upper positive charge. This may explain how the narrow upper positive charge region found in Fig. 2c is formed. This hypothesis is supported by the electrical measurements made at high altitudes by U-2 airplanes above thunderstorms with high cloud tops^37,38^. These measurements show the vertical component of the electric field becomes increasingly positive as the airplane passes above the highest cloud tops of the thunderstorm. This suggests (for a normal polarity storm) the upper negative screening charge is being pushed to the sides of the overshooting top, creating a ‘hole’ in the screening charge layer, which reveals the upper positive charge (see Fig. 9 of Vonnegut *et al*.^37^). Mixing of upper negative screening charge with upper positive charge near high cloud tops has been hypothesized before, from observations of anomalous VHF activity in the upper regions of thunderstorms^39–43^. The authors of those studies speculated that this was caused by the upper negative screening layer being folded into the top of the thunderstorm^41–43^. This motion near cloud top is similar to the entrainment studies described by Blyth *et al*.^44^ and Stith^45^.

The findings reported here suggest that convective pulses creating overshooting tops are a primary driver in creating the charge structures that produce negative gigantic jets. However, convective pulses and overshooting tops are commonly found in supercell and multicell convection throughout the mid-latitudes, where gigantic jet observations are infrequent. The question is then: why are there not more gigantic jet observations from mid-latitude convection where intense updrafts and overshooting tops are commonplace? The answer to this question is likely related to the differences between the charge structures of mid-latitude and maritime tropical convection that have intense, pulsating updrafts that produce overshooting tops. Our limited results may be suggestive that (also see Supplementary Information) maritime tropical convection with pulsating overshooting tops exhibit relatively simple charge structures similar to the normal tripolar configuration with upper positive charge, middle negative charge, and possibly a small lower positive charge. This is supported by the dominant positive IC and negative CG discharges from gigantic jet producing storms^6–8^. In contrast, the charge structures in supercell convection have been found to be very complex, with anywhere from three to twelve charge regions existing simultaneously. These charge regions are often adjacent to each other, which results in small intracloud flashes that occur at very high rates, with total flash rates in supercell convection often reaching several hundred per minute^40,42,46,47^. Total flash rates from gigantic jet producing convection have been found to be an order of magnitude lower^6–8^ or even less. Mid-latitude supercell and multicell convection often have anomalous charge structures that have huge areas of middle/lower positive charge^48–50^. These storms exhibit large percentages of positive CG discharges (50–100% compared with <10% for normal convection^51^) and IC discharges between the middle negative and middle/lower positive charge regions (negative IC discharges). For these anomalously charged storms, the middle/lower positive charge participates in the majority of discharges, so few discharges take place in the upper parts of the thundercloud. Thus, for mid-latitude supercell and multicell convection that have intense updrafts and overshooting tops, other forms of discharges often win the competition to neutralize charge, such as discharges between adjacent pockets of charge and negative IC discharges, rather than the normal positive IC discharge between vertically stacked regions of upper positive and middle negative charge that is associated with the initiation of gigantic jets^14–16^.

## Methods

### Radar and Lightning Analysis

Radar data was obtained from the dual-polarization Weather Surveillance Radar 88-Doppler network from the KMLB (Melbourne, FL), KAMX (Miami, FL), and KINX (Tulsa, OK) radar sites. By analyzing the radar reflectivity, radial velocity, and spectrum width near the upper regions of the thundercloud, insight into the storm structure near the upper positive charge region was obtained. The VHF lightning data was from the Oklahoma Lightning Mapping Array^52^ and the Kennedy Space Center Lightning Detection and Ranging (KSC LDAR) network^52,53^. The charge analysis in Fig. 2 was completed by using characteristics inherent to how the VHF networks detect leader breakdown in positive or negative charge regions^47,54^ and also by analyzing the leader speeds^55^. The leader speeds observed during the analysis times were typical for negative leaders (10^5^ m s^−1^) and positive leaders (10^4^ m s^−1^)^55,56^. The specific periods analyzed in Table 2 and Fig. 2 correspond to intervals when the storm was undergoing rapid changes as identified by the VHF charge structure and radar data. One minute time intervals were chosen due to the rapid variation of the charge structure, as observed by the VHF data.

Due to the smaller azimuthal uncertainty of the VHF network^52^, the vertical cross sections in Fig. 2 were taken along the azimuthal direction. The statistics in Table 1 were completed by projecting the VHF points onto a plane perpendicular to a radial path extending from the VHF network. The value of ΔX was found by taking the one standard deviation *σ*_*x*_ about the mean in the azimuthal direction. The mean altitude was found by taking the mean of the VHF points in the vertical direction. The NLDN events associated with each gigantic jet were chosen as the closest positive IC event (negative charge moving upward) to each gigantic jet in space and time. Figures S1 and S2 and Table S1 were similarly created this way. However, considering the VHF mapping systems detected fewer points due to the large radial distance from the mapping systems and their respective thunderstorms, the analysis times only included points associated with the upper positive charge. Signatures of the positive leaders in the middle negative charge region were sparse and not included in Figures S1 and S2. Also, the mean altitude of the VHF points was not calculated, as the points had a large variation in the vertical direction.

### Lightning Simulation Model

The three dimensional probabilistic fractal model used in this study has been described in detail in other studies^14,15,24^. The simulations presented here used equidistant grid points of 400 m in the x, y, z directions and used open boundaries^24^ over a perfectly electrically conducting flat ground plane with zero potential. Simulations with a smaller or larger grid size gave similar results (see Fig. S5). When a smaller (larger) grid size was used, the net amount of negative charge needed to form an escaped leader was less (more). This is due to the leader trees with fine resolution being able to occupy the regions of thundercloud charge better than the leader trees with coarse resolution, which resulted in more charge on the leader tree for the fine resolution cases (this effect of the grid resolution was noted previously by Mansell *et al*.^23^). The simulation also uses an internal channel field of 1.0 kVm^−1^ to account for leader resistivity^57^, which is similar in magnitude to other lightning modeling studies^23^. The internal electric field is assumed to scale with neutral density as $\frac{N}{{N}_{0}}$, as it has been shown that the reduced electric field in the channel formed after streamer-to-leader transitions is about the same at 20 km and 40 km altitudes (Fig. 16 of da Silva and Pasko^58^), but it should be noted that the scaling law of the leader channel field has not been well studied. A larger vertical domain was also used (30 km altitude) compared to previous simulations of gigantic jets^14,15^. The top boundary of the simulation (terminal altitude) was chosen because the model can only simulate a conducting leader channel, and the gigantic jet is predominantly composed of leaders below this altitude^58^. The simulation with lower positive charge (Fig. S4) required more middle negative charge for the leader to escape.

The constraints to determine the amount of charge in each region were (**1**) the leader discharge is initiated at the location where the electric field exceeds by 1–10% of the threshold value of the ambient field for leader initiation (about 200 kVm^−1^ at ground pressure)^26–29^ (**2**) the charge densities must be less than the maximum values found from observations^30–33^ (a few tenths to a few nCm^−3^), and (**3**) the charge structures must have the minimum amount of net negative charge in the thundercloud for a negative leader to escape.

Each charge region has a Gaussian distribution. The charge structures in Fig. 3a,d,g all have middle negative charge regions centered at 7.5 km altitude, with a full width half maximum (FWHM) of 10 km in horizontal and 2 km in the vertical direction. The upper positive charge in Fig. 3a,d,g is centered at 13 km altitude. The FWHM of the upper positive charge in Fig. 3a,d is 5 km in the horizontal and 2.15 km in the vertical direction. The negative screening charge is centered at 14.0 km altitude, has a FWHM of 10 km in the horizontal and 0.80 km in the vertical direction. The ‘hole’ in the screening charge is created by slightly penetrating the narrow upper positive charge into the large wide screening layer. Lastly, the upper positive charge in Fig. 3g has a FWHM of 12 km in the horizontal and 2 km in the vertical direction.

## Electronic supplementary material

Supplementary material
