# Supplementary material for: Thunderstorm charge structures producing gigantic jets
Source: Sci Rep. 2018 Dec 27;8:18085. doi: 10.1038/s41598-018-36309-z (PMC6308230; doi:10.1038/s41598-018-36309-z)
Supplement: Supplementary file 1 — Supplementary material [file 41598_2018_36309_MOESM1_ESM.pdf]

Supplementary Material for 'Thunderstorm charge structures producing gigantic jets'

L. D. Boggs, N. Y. Liu, J. A. Riousset, F. Shi, S. M. Lazarus, M. E. Splitt and H.K. Rassoul

FIG. S1. **Evolution of the upper positive charge region for the gigantic jets from Tropical Depression Dorian on 03 August 2013**. VHF inferred charge structure for the (a) gigantic jet time 1 (b) gigantic jet time 2 (c) post pulse time 1 and (d) post pulse time 2. The white circles denote upper positive charge as inferred from the VHF mapping system. The red vertical lines in (a),(b) denote the edges of large spectrum width values shown in Fig. 1c, column 3. Distance scales are listed in km.

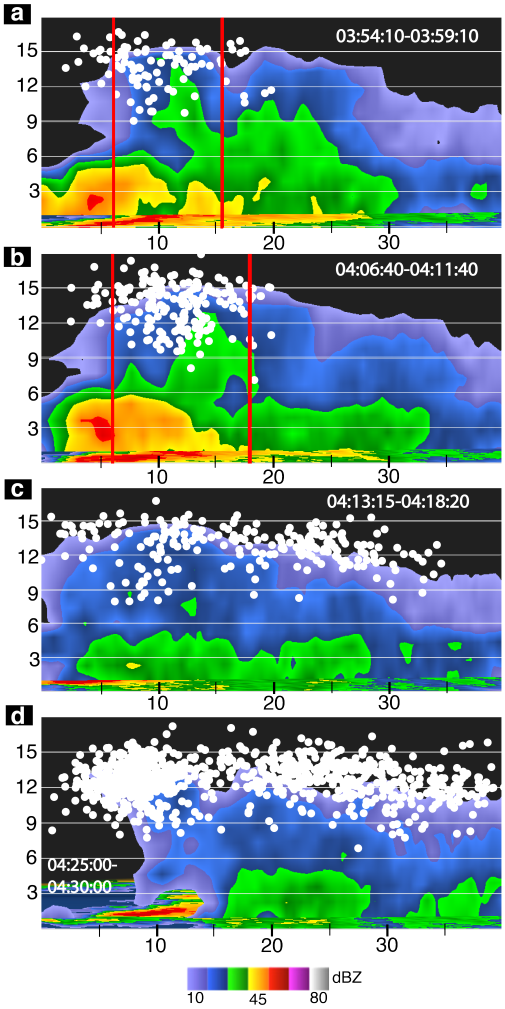

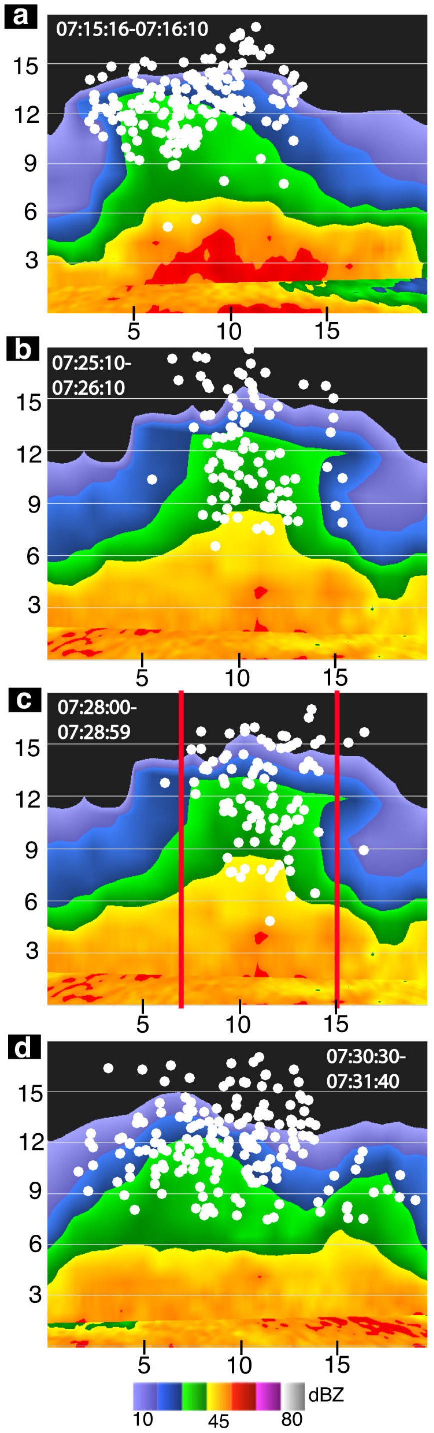

FIG. S2. **Evolution of the upper positive charge region for the Oklahoma gigantic jet on 09 September 2010 at 07:28:20 UTC**. VHF inferred charge structure for the (a) pre-pulse (b) initial pulse (c) final pulse (gigantic jet) and (d) post pulse times. The white circles denote upper positive charge as inferred from the VHF mapping system. The red vertical lines in (c) denote the edges of large spectrum width values shown in Fig. 1b, column 3. Distance scales are listed in km.

OK 2010	Time (UTC)	ΔX (km)
Pre-Pulse	07:15:16-07:16:10	6.4
Initial Pulse	07:25:10-07:26:10	4.3
Final Pulse (GJ)	07:28:00-07:28:59	4.3
Post-Pulse	07:30:30-07:31:40	7.8
**FL 2013**		
Gigantic Jet 1	03:54:10-03:59:10	4.3
Gigantic Jet 2	04:06:40-04:11:40	3.8
Post-Pulse 1	04:13:15-04:18:20	8.9
Post-Pulse 2	04:25:00-04:30:00	10.6

TABLE S1. **Statistics describing the upper positive charge region for the Oklahoma thunderstorm on 09 September 2010 and the Florida thunderstorm on 03 August 2013.** ΔX represents one standard deviation about the mean of the azimuthal VHF sources.

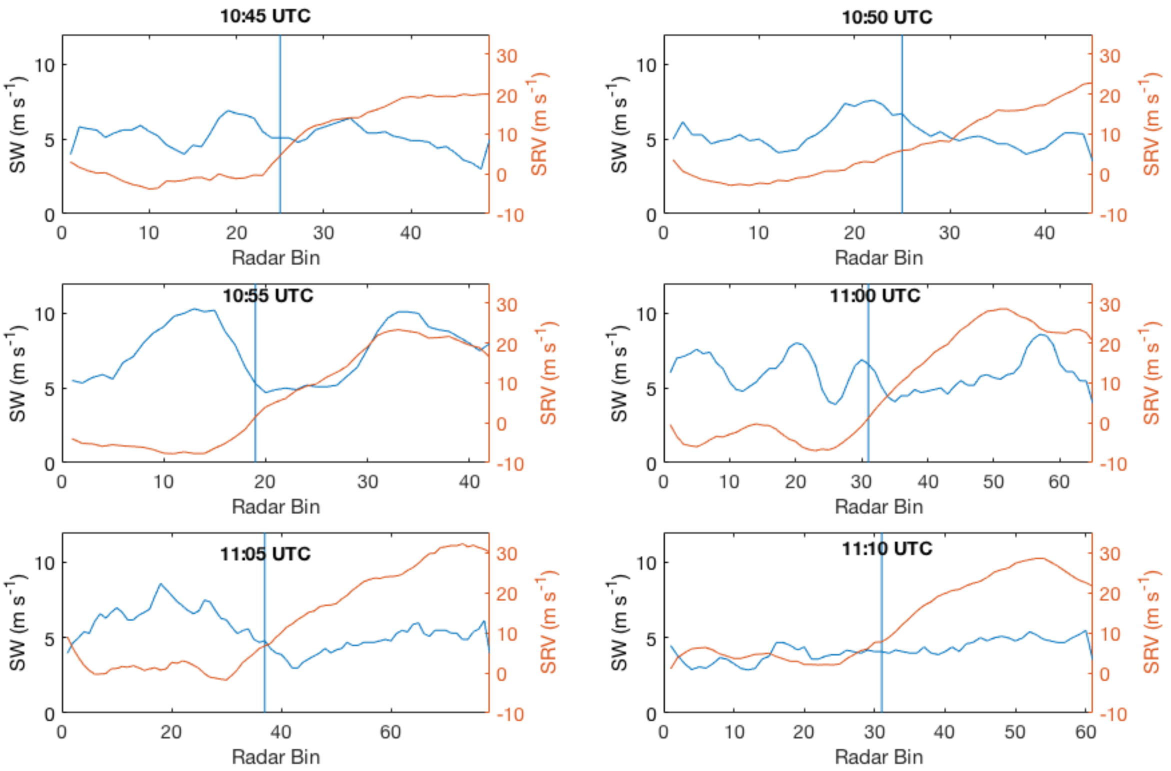

FIG. S3. **Evolution of storm top radial velocity and spectrum width for storm on 28 September 2010**. Spectrum width (SW) and Storm Relative Velocity (SRV) along a radial through the largest reflectivity at storm top. The radar bin numbers on bottom axis do not all begin at the same location, but are centered around the convective core for each time. The center line represents the center pixel of reflectivity $>$ 30 dBZ.

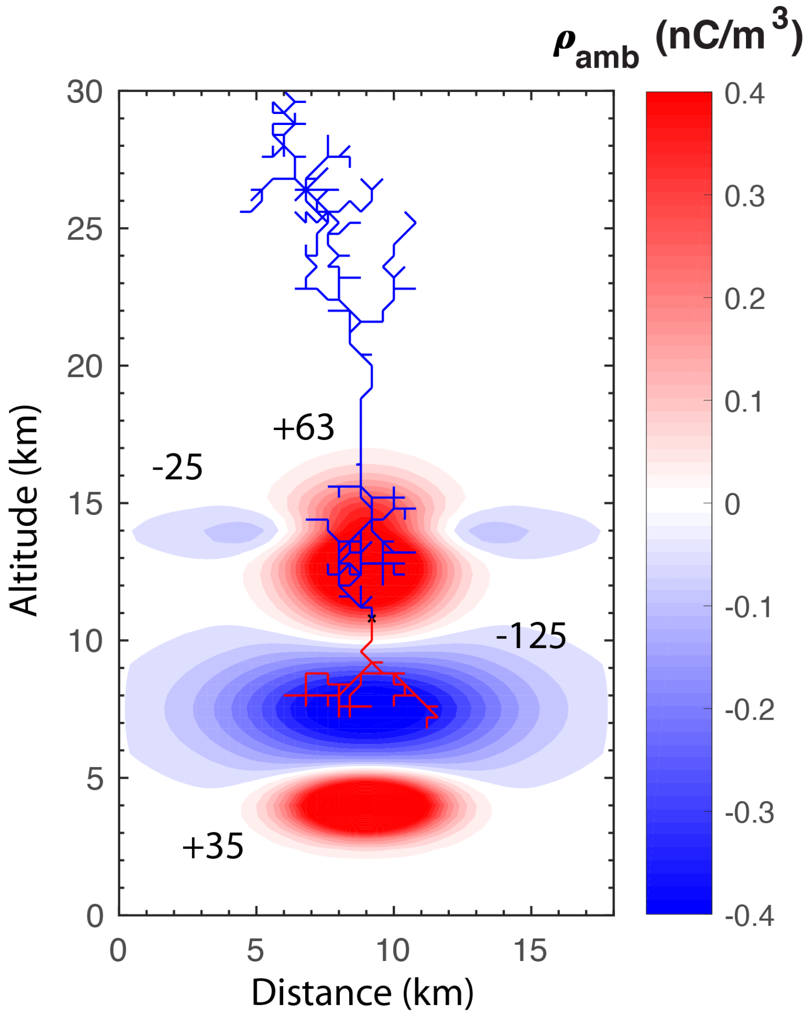

FIG. S4. **Modeling result with lower positive charge**. Simulated discharge trees overlaid on Gaussian thunderstorm charge structures for a charge structure with narrow upper positive charge, wide middle negative charge, upper screening charge, and lower positive charge. Positive (negative) charges and leaders are colored in red (blue). Charge amounts are in Coulombs. 'X's denote location of discharge initiation.

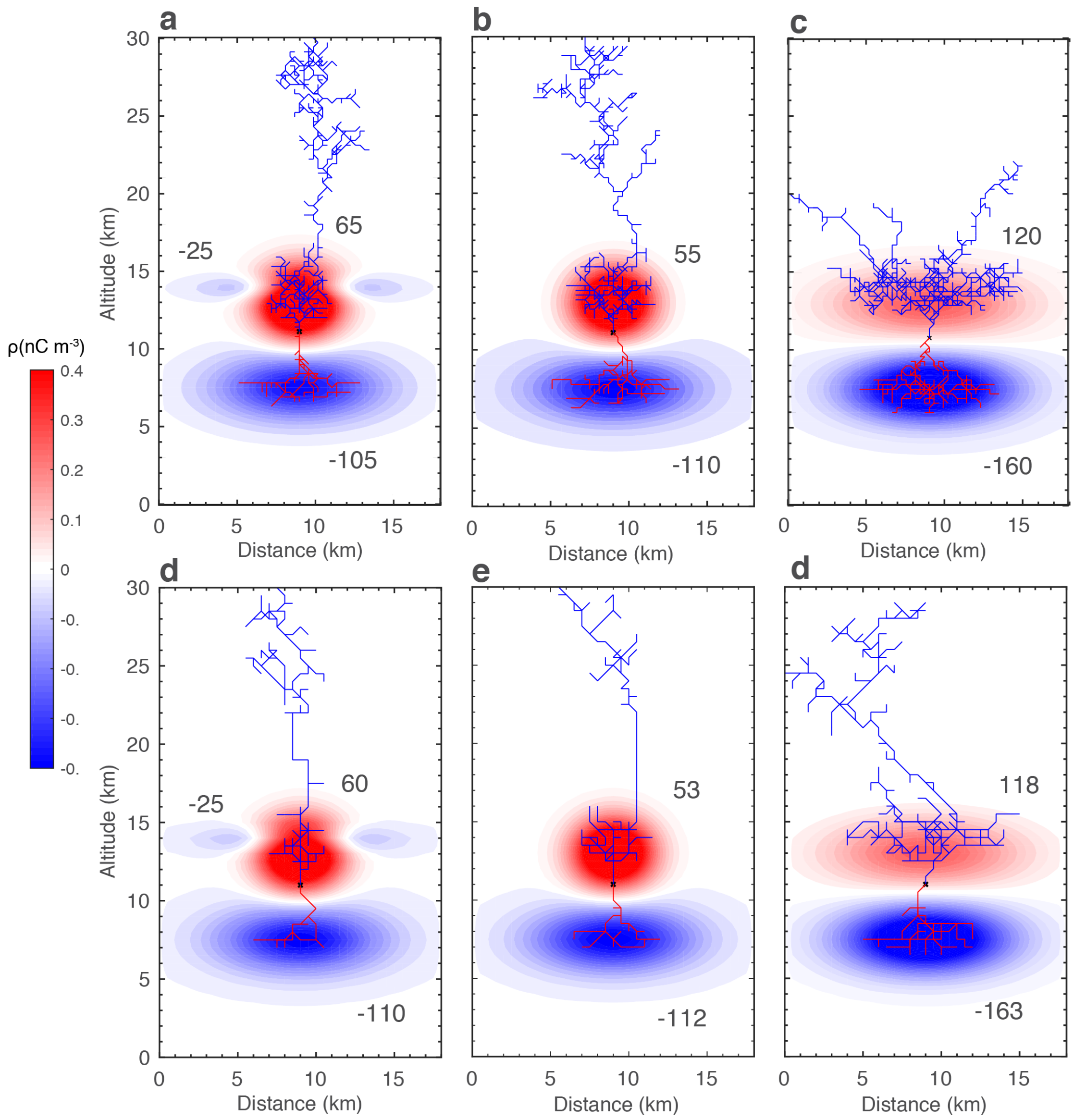

FIG. S5. **Modeling results with different grid sizes**. Simulated discharge trees overlaid on Gaussian thunderstorm charge structures for (a-c) 300 m and (d-f) 500 m grid resolution. Positive (negative) charges and leaders are colored in red (blue). Charge amounts are in Coulombs. 'X's denote location of discharge initiation. Distance scales are listed in km.
